# A potential herbal therapeutic for trichinellosis

**DOI:** 10.3389/fvets.2022.970327

**Published:** 2022-08-23

**Authors:** Asmaa M. El-kady, Iman A. M. Abdel-Rahman, Eman Sayed, Majed H. Wakid, Hussah M. Alobaid, Khalil Mohamed, Eman Abdullah Alshehri, Hayam Elshazly, Wafa Abdullah I. Al-Megrin, Furhan Iqbal, Hatem A. Elshabrawy, Ashraf G. Timsah

**Affiliations:** ^1^Department of Medical Parasitology, Faculty of Medicine, South Valley University, Qena, Egypt; ^2^Department of Pharmacognosy, Faculty of Pharmacy, South Valley University, Qena, Egypt; ^3^Department of Parasitology, Faculty of Veterinary Medicine, South Valley University, Qena, Egypt; ^4^Department of Medical Laboratory Technology, Faculty of Applied Medical Sciences, King Abdulaziz University, Jeddah, Saudi Arabia; ^5^Special Infectious Agents Unit, King Fahd Medical Research Center, Jeddah, Saudi Arabia; ^6^Department of Zoology, College of Science, King Saud University, Riyadh, Saudi Arabia; ^7^Department of Epidemiology, Faculty of Public Health and Health Informatics, Umm Al-Qura University, Mecca, Saudi Arabia; ^8^Department of Biology, Faculty of Sciences-Scientific Departments, Qassim University, Buraidah, Saudi Arabia; ^9^Department of Zoology, Faculty of Science, Beni-Suef University, Beni Suef, Egypt; ^10^Department of Biology, College of Science, Princess Nourah bint Adbulrahman University, Riyadh, Saudi Arabia; ^11^Zoology Division, Institute of Pure and Applied Biology, Bahauddin Zakariya University, Multan, Pakistan; ^12^Department of Molecular and Cellular Biology, College of Osteopathic Medicine, Sam Houston State University, Conroe, TX, United States; ^13^Department of Microbiology, Faculty of Medicine, Al-Baha University, Al Baha, Saudi Arabia; ^14^Department of Parasitology, Faculty of Medicine, Al-Azhar University, New Damietta City, Egypt

**Keywords:** *Artemisia annua*, *T. spiralis*, goblet cells, TGF-β, trichinellosis

## Abstract

**Background:**

Trichinellosis is a helminthic disease caused by *Trichinella spiralis via* the ingestion of raw or undercooked meat of infected animals. Current estimates indicate that 11 million humans have trichinellosis, worldwide. The effective use of anti-trichinella medications is limited by side effects and resistance which highlight the critical need for safe and effective drugs, particularly those derived from medicinal plants. Therefore, in the present study, we aimed to evaluate the efficacy of the ethanolic extract of *Artemisia annua* (*A. annua*) in treatment of experimentally induced trichinellosis.

**Materials and methods:**

Trichinellosis was induced experimentally in male 6–8 weeks BALB/c mice. BALB/c mice were divided into four groups, 10 mice each. One group was left uninfected and untreated, whereas three groups were infected with *T. spiralis. One* infected group of mice was left untreated (negative control) while the remaining two infected groups received either 300 mg/kg of the ethanolic extract of *A. annua* or 50 mg/kg of albendazole (positive control). All treatments started from the third day post-infection (dpi) for 3 successive days. All animals were sacrificed on the 7th dpi for evaluation of treatment efficacy.

**Results:**

Our findings showed that *A. annua* treatment reduced the *T. spiralis* adult-worm count in the intestine of infected animals. Moreover, treatment with *A. annua* restored the normal intestinal architecture, reduced edema, alleviated inflammation as demonstrated by reduced inflammatory infiltrate and expression of TGF-β in intestinal tissues of *A. annua*-treated animals compared to infected untreated animals.

**Conclusions:**

Our findings show that *A. annua* extract is effective in treating experimentally induced trichinellosis which highlight the therapeutic potential of *A. annua* for intestinal trichinellosis.

## Introduction

Trichinellosis is a helminth disease caused by *Trichinella spiralis* nematode (*T. spiralis*). Ingestion of the raw or undercooked meat of infected animals is the major source for the transmission of *T. spiralis* that have infected around 11 million humans globally (1). In 2014, the Food and Agriculture Organization of the United Nations (FAO), and the World Health Organization (WHO) had listed *T. spiralis* among the top 10 most common foodborne parasites that eventually lead to serious health problems. Trichinellosis have become a public health concern and is considered a re-emerging disease with recent outbreaks reported in 55 countries (2–6).

The early clinical signs of trichinellosis are gastrointestinal problems, such as abdominal pain, diarrhea, nausea, and vomiting, which commonly start 2–7 days following consumption of raw or undercooked meat (7). These manifestations are due to invasion of intestinal epithelial cells by *T. spiralis* larvae where they grow to adults, mate and produce neonatal larvae 3–7 days post infection (dpi) (7). Studies have shown that adult *T. spiralis* were expelled from the intestine within 10–17 days following infection which indicates that the intestinal phase (the first 17 dpi) is considered the critical trichinellosis stage which determines the progression and outcome of the disease (8, 9).

*T. spiralis* adult worms have been shown to trigger acute inflammatory responses resulting in a series of intestinal pathological changes (10). Previous studies have shown that Th2, dendritic cells (DCs), eosinophils, and mast cells are the key cells involved in immune responses against *T. spiralis*. A strong Th2 response was demonstrated in *T. spiralis* infection which eventually lead to a transient inflammatory reaction and worm expulsion (11–14). Th2 immune response is mediated by soluble mediators such as IL-4, IL-5, IL-9, IL-13 which in addition to histamine, released from mast cells, and antibodies (IgE and IgG1) result in adult worm expulsion (15, 16). Although, the expulsion mechanisms depend mainly on Th2 cytokines; however, the pathways and mechanisms responsible for this process remain unclear (15, 16).

To date, albendazole and mebendazole are the main anthelmintic medications used for treatment of trichinellosis (17). However, their therapeutic utility is limited by emerging resistance and the reported side effects such as acute liver injury, anemia, leukopenia, hypercholesterolemia, proteinuria, hemolytic anemia, and, in rare occasions, kidney and brain injuries (18–22). Moreover, safety for pregnant women and children under the age of 3 years remains a concern (23). Therefore, the development of safe and effective anti-trichinellosis drugs is a necessity (24).

In traditional medicine, medicinal plants have long been used. *Artemisia annua (A. annua)*, a plant known for its malarial properties, has been researched for a variety of biological activities, including anticancer, antidiabetic, and antibacterial properties (25–32). In addition to treatment of several parasitic diseases such as giardiasis (33), toxoplasmosis (34) and leishmaniasis (35). *A. annua* contains a wide range of chemical compounds (36, 37). Many bioactive compounds have been isolated from this plant; however, artemisinin is the most active of these compounds (38–40). Other important constituents in *A. annua* include specific sesquiterpene lactones, essential oils (41), tannins, saponins (42), polyalkenes (43), phytosterols, fatty acids (42), and proteins (42).

In the present study, we evaluated the efficacy of the ethanolic extract of *A. annua* in treatment of BALB/c mice, that were experimentally infected with *T. spiralis*. Based on parasitological and histopathological analysis, we report high efficacy of *A. annua* in treatment of intestinal trichinellosis. Our results indicate that *A. annua* ethanolic extract is a potential natural therapeutic for trichinellosis.

## Materials and methods

### Preparation of *A. annua* extract

Leaves of *A. annua* were obtained during the flowering stage from a farmed field near South Valley University, Qena, Egypt. Plant was identified by Prof. Naglaa Lofty; an associate professor of Botany at the Faculty of Science, South Valley University, Qena, Egypt. The plant's voucher specimen (code: Aa.78) was kept in the herbarium of the Department of Pharmacognosy, Faculty of Pharmacy, South Valley University, Qena, Egypt following their taxonomic identification. The freshly collected plant was allowed to dry at room temperature before being grounded into fine powder. *A. annua* ethanolic extract was then prepared as previously described (44). Briefly, 200 g of powder was macerated in 1L of 95% ethanol at 25°C for 16h, filtered, and the filtrate was dried in a rotary evaporator at 40°C under reduced pressure (45). The dried extract was stored at 4°C till used in the preparation of 300 mg/kg dose for mice experiments (46, 47). The *reference drug* albendazole was obtained as a suspension (20 mg/mL) from the Egyptian International Pharmaceutical Industries Co, Cairo, Egypt.

### Preparation of infective *T. spiralis* larvae

BALB/c mice infected with *T. spiralis* were obtained from the Faculty of Medicine, Assiut University, Assiut, Egypt. Larvae were obtained from the infected muscles as previously described (48). Briefly, infected muscles were digested by immersion in a digestive solution (1,000 ml saline containing 20 mL of concentrated HCl and 20 g of Pepsin) for 12 h at 37°C on a mechanical stirrer. To release the larvae, the suspension was centrifuged at 1,000 rpm for 2 min. Material was rinsed with normal saline (0.9% NaCl) and was re-centrifuged. The larvae were counted by using hemocytometer to determine the inoculum size that required to infect mice. The sediment containing the larvae was then re-suspended in saline containing 1.5% gelatin for their use in the animal experiments.

### Experimental design

Parasite free 6–8-week-old male BALB/c mice (*N* = 40), weighing 25–30 g, were purchased from Theodor Biharz Research Institute, Giza, Egypt, and were kept in a sanitary condition in Faculty of Veterinary Medicine, South Valley University, Qena, Egypt during the whole experiment. Mice were housed under controlled temperature and light conditions with water and standard rodent diet, *ad libitum*.

Mice were divided into four groups, 10 mice each. Group A represent uninfected mice (negative control) while Groups B, C, and D were starved for 12 h then orally infected, each with 300 *T. spiralis* larvae, using a syringe (24). Group B did not receive any treatment (infected untreated mice), whereas Group C and D infected mice were treated with 50 mg/kg albendazole (49) and 300 mg/kg of the *A. annua* ethanolic extract, respectively (18, 33). All treatments started from the third day post-infection (3 dpi) for three consecutive days (18, 50). All animals were sacrificed on the 7th dpi for the evaluation of treatment efficacy (24, 49, 51).

### Assessment of the therapeutic efficacy of *A. annua* extract in intestinal trichinellosis

#### Isolation and counting of *T. spiralis* adult worms in the intestines of different mice groups

*T. spiralis* adult worms were isolated from the small intestine of each mouse and counted as previously described by Denham (52). The small intestine of each animal was split longitudinally, cut into 5 cm pieces and incubated in 0.9% saline for 2h at 37°C (53). Subsequently, intestine was incubated in sodium hydroxide (0.05%) for 6h at 5°C. The worms were then retrieved by washing the intestines with water and passing the wash through *via* a 200-mesh sieve (52). The collected material was washed with distilled water and streaked across 2- × 3-inch slides by using a Pasteur pipette. Total number of worms in all intestinal 5 cm pieces/animal were counted under a dissecting microscope and the mean number of worms/group was calculated for comparison between different groups.

#### Histopathological examination of small intestine of mice from different groups

##### Hematoxylin and eosin staining

For detection of histopathological changes in different animal groups, we used 1 cm specimens from the junction of the proximal (1/3) and distal (2/3) parts of the intestine of mice sacrificed on the 7th dpi. The intestinal specimens were fixed in 10% formalin and embedded in paraffin (54). Sections, with a 3 mm thickness, were stained with hematoxylin and eosin (H&E) as previously described (54, 55) and evaluated by an independent blinded pathologist. Histopathological changes of the intestinal tissue were used to assess the intensity of inflammation and were scored from 0 to 4 (0, normal; 1, mucosal hyperplasia; 2, spotty infiltration by inflammatory cells not involving the entire mucosa and/or submucosal thickness; 3, marked increase of inflammatory cells involving the full thickness of the mucosa and/or submucosal thickness; 4, marked increase of inflammatory cells in both the mucosa and submucosa).

Villi length was measured from the tip of villi to their base at the mucosal surface. Crypt depth was measured from the base of villi up to the beginning of the muscularis mucosa. For the detection of eosinophils, at least three sections/animal were examined under light microscopy at 400X magnification.

##### Determination of goblet cell numbers in the intestinal mucosa

We used fixed 2 cm sections of the duodenum, jejunum, and ileum with Carnoy's solution. The tissues were embedded in paraffin, cut with a microtome, and stained with Periodic Acid Schiff (PAS) stain. Hematoxylin was used as the counterstain. The number of goblet cells in mucosal linings in 3 high power fields (HPF; 400X) were counted, and the mean number ± S.D was calculated (56).

##### Immunohistochemistry

Small intestine sections embedded in paraffin were cut into 4 μm thick sections for immunohistochemical (IHC) staining. Sections were de-paraffinized and rehydrated with descending ethanol grades. Sections were placed in citrate buffer (pH 6.0) and heated in microwave for epitope retrieval. Endogenous peroxidases were then blocked by using 3% H_2_O_2_ in ethanol (for 10 min) and incubated with the primary anti-TGF-β rabbit polyclonal antibody (ABclonal USA, Catalog No.: A16640) for 1h at room temperature. Sections were washed with TBS containing 0.05% Tween-20 (TBS-T) and incubated with HRP-conjugated goat anti-rabbit secondary antibody for 1h at room temperature (Abcam, Cambridge, MA, USA. Catalog No.: HRP ab6728). Finally, slides were washed with TBS-T and the color was developed by incubating the tissue sections with 0.05% diaminobenzidine (DAB) and 0.01% H_2_O_2_ for 3 min. The sections were finally counterstained with hematoxylin for 1 min prior to dehydration and mounting. Sections with no added primary antibody were used as negative control. Images were captured using an Olympus light microscope equipped with a digital camera (Olympus, Japan, BX53). The number of TGF-β-positive mucosal glandular and stromal cells, and submucosal stromal cells were counted in 10 images captured at 400x and their percentage were calculated (57).

### Statistical analysis

The results were analyzed using the Statistical Package for Social Sciences (SPSS) version 20 for Windows. All values are expressed as mean ± Standard deviation (SD). ANOVA test was used for the comparison of mean number of adult worms, goblet cells and eosinophils in the intestine wall between all groups of animals. *P*-values < 0.05 were considered statistically significant.

## Results

### Treatment with *A. annua* extract effectively reduced the number of *T. spiralis* adult worms in the intestine of infected animals

**Figure 1:**
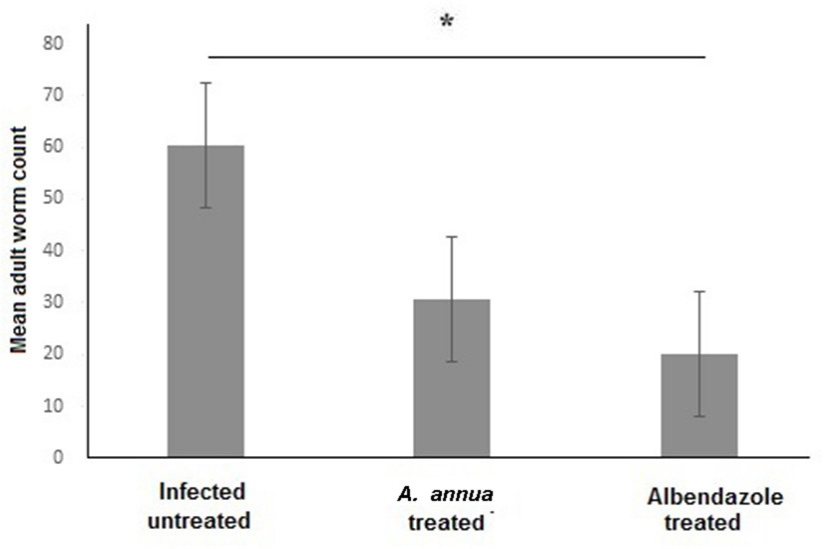
Effect of *A. annua* treatment on the number of adult *T. spiralis* worm in the small intestine of infected mice. Treatment with *A. annua* extract significantly reduced worm count in the intestine of infected mice, an effect that was comparable to albendazole (positive control). Data are expressed as mean ± SD (*N* = 10) and analyzed by using ANOVA followed by LSD as a *post hoc* test. Asterisk (*) indicate significant difference; *p* < 0.05.

We aimed to evaluate the anthelmintic efficacy of *A. annua* extract against *T. spiralis* adult worms in the intestine of infected mice. Examination of the small intestines of *A. annua* and albendazole-treated animals (positive control) revealed a significant reduction in the *number of T. spiralis* adult worms compared to infected untreated animals (*P* = 0.004) (Figure 1). However, there was no significant difference between the number of adult worms in *A. annua* and albendazole-tre*at*ed mice (*P* = 0.19). Our results indicated that *A. annua* extract is effective in reducing the number of *T. spiralis* adult worms in the small intestine of infected mice.

### *A. annua* treatment protected the intestine against infection-induced pathological changes

**Figure 2:**
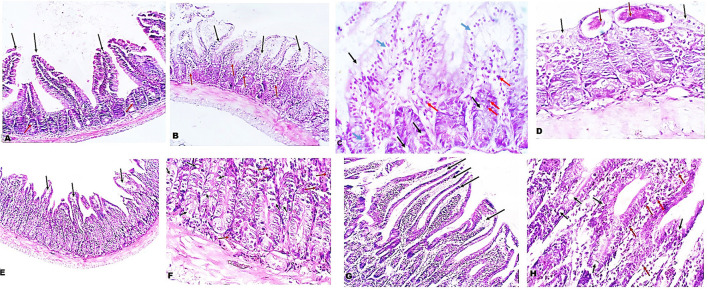
Treatment with *A. annua* restored villi architecture and alleviated inflammation in intestinal tissues of *T. spiralis*-infected mice. **(A)** Representative image of small intestinal tissue of uninfected mice stained with H&E showing uniform villi and goblet cells (100x). **(B)** Representative image of small intestinal tissue of *T. spiralis*-infected untreated mice showing distorted degenerated edematous villi (black arrows) with significant mucosal inflammation (red arrows) (100x). **(C)** Higher magnification of **(B)** (400x) demonstrating edema in villi and lamina propria (blue arrows) and also high goblet cell numbers (black arrows) and inflammatory infiltrate composed mainly of eosinophils (red arrows) **(D)** Higher magnification of **(B)** (400x) with parasite on surface (red arrows) of distorted villi (black arrows). **(E)** Representative image of small intestinal tissue of infected mice treated with *A. annua* extract showing restoration of villi outline and reduced edema (black arrows). (**F)** Higher magnification (400x) of **(E)** with similar number of goblet cells (black arrows) and lower inflammatory infiltrate than infected untreated mice (red arrows). **(G)**. Representative image of small intestinal tissue of infected mice treated with albendazole showing protected villi and reduced edema (black arrows) compared to infected untreated mice. **(H)** Higher magnification (400x) of **(G)** showing reduced inflammatory infiltrate (red arrows) and similar frequency of goblet cell number (black arrows) compared to infected untreated mice.

Next, we examined the intestinal sections of different mice groups to evaluate the therapeutic effect of *A. annua* in reducing the pathological changes due to *T. spiralis* infection. H&E staining of intestinal sections of uninfected untreated mice showed long regular, finger-like intestinal villi (black arrows), shallow intestinal crypts, and sparse goblet cells (red arrows) (Figure 2A). However, intestinal sections of infected untreated mice showed shortened, leaf-shaped, degenerated, distorted and edematous intestinal villi with some areas showing complete villous atrophy (black arrows) and significant mucosal inflammation (red arrows) (Figure 2B). Higher magnification (400x) showed edema in villi cores and lamina propria (blue arrows) and increase in goblet cell numbers (black arrows) (Figure 2C). Moreover, inflammatory cellular infiltrate was detected in the lamina propria composed mainly of eosinophils (red arrows). *T. spiralis* adult worms were also observed (red arrows) lying on surface of distorted intestinal villi (black arrows) (Figure 2D). Conversely, the intestinal sections of mice treated with *A. annua* extract showed marked improvement of the villi outline along with reduced edema (black arrows) and absence of worms (Figure 2E). Higher magnification (400x) demonstrated similar frequency of goblet cells (black arrows) but reduced inflammatory infiltrate (red arrows) (Figure 2F) compared to infected untreated mice. Similarly, albendazole treatment preserved villi structure and length with mild edematous cores (black arrows) (Figure 2G), while reducing inflammatory cellular infiltrates (red arrows) with no significant change in numbers of goblet cells compared to infected untreated mice (black arrows) (Figure 2H).

**Figure 3:**
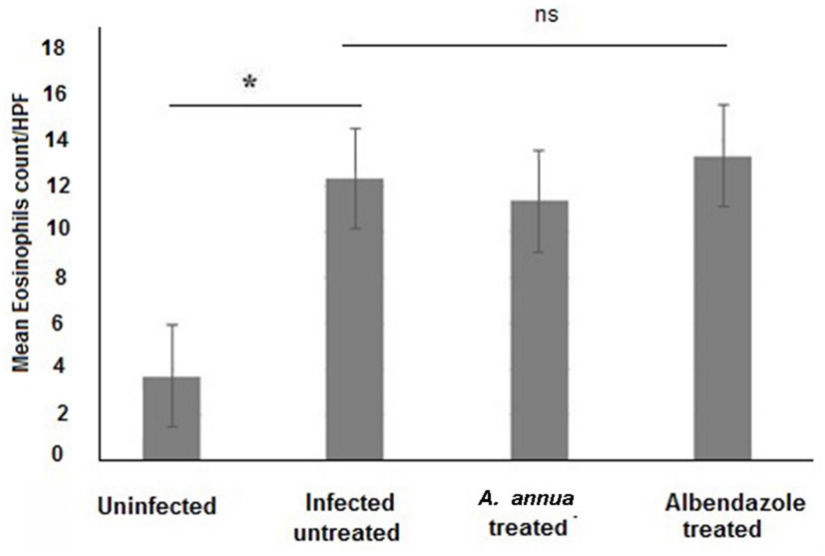
Eosinophil cell numbers in intestinal tissues of different groups of mice. Infection of mice with *T. spiralis* significantly increased eosinophil cell number in intestinal tissue/high power field (HPF) compared to uninfected mice. Treatment with either *A. annua* or albendazole (positive control) did not result in significant change (ns) in eosinophil cell numbers infiltrating the mucosa and submucosa of small intestine compared to infected untreated mice. Asterisk (*) indicate significant difference between uninfected and infected untreated mice (*p* < 0.007), whereas ns, non-significant.

**Figure 4:**
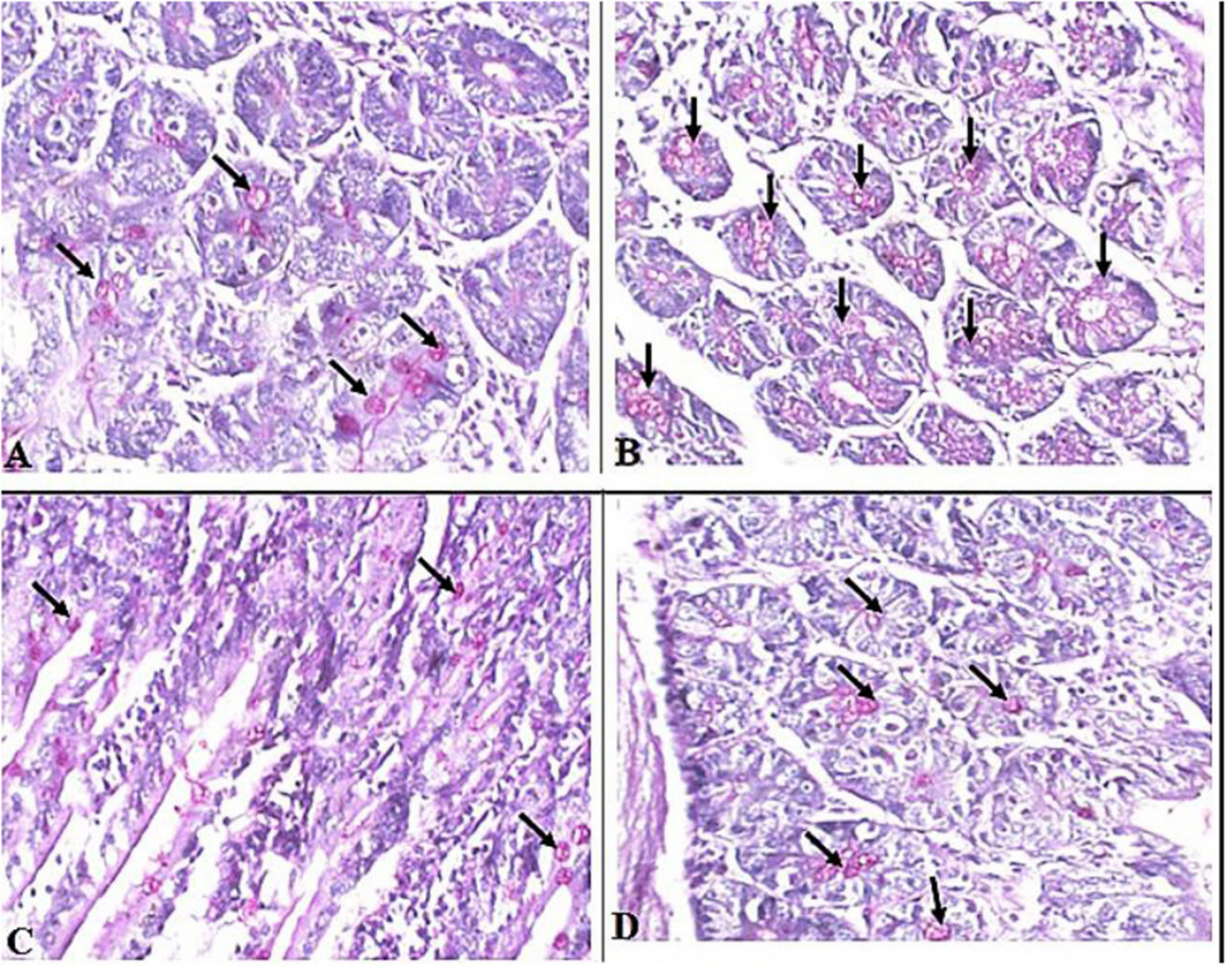
Representative microphotographs of intestinal tissue of different groups of mice showing goblet cells. Periodic Acid Schiff's (PAS) staining was performed to demonstrate goblet cells in intestinal tissues. **(A)** Uninfected mice. **(B)** Infected untreated mice. **(C)** Intestinal tissue of mice treated with *A. annua* ethanolic extract. **(D)** Intestinal tissue of mice treated with albendazole (positive control). Black arrows point to goblet cells. Magnification, 400x.

**Figure 5:**
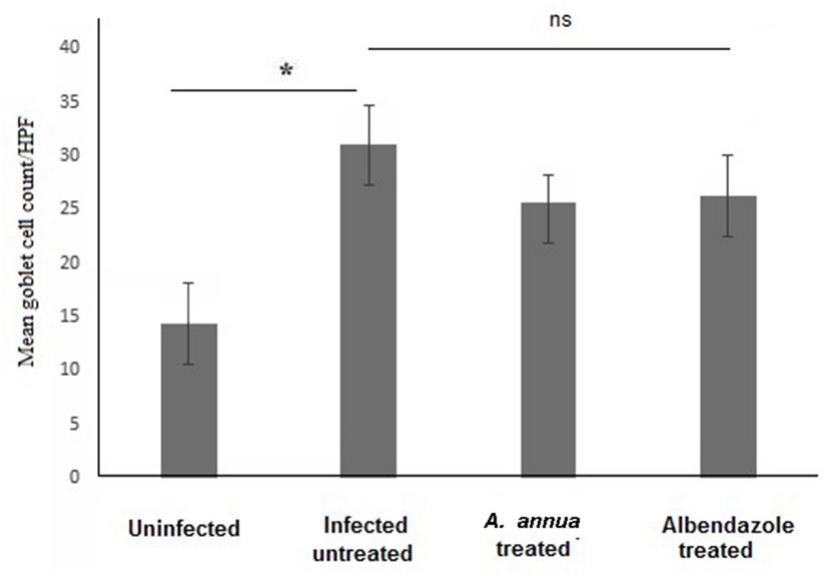
Goblet cell numbers in intestinal tissues of different groups of mice. High magnification images (400x) of intestinal tissues stained with PAS and hematoxylin were used to count goblet cells. All infected mice groups (treated and untreated) showed significant high numbers of goblet cells/HPF in comparison to uninfected mice group. Asterisk (*) indicate significant difference, whereas ns, non-significant.

Interestingly, we found that eosinophils were significantly elevated in all *T. spiralis*-infected mice compared with uninfected mice (*P* = 0.007) (Figure 3). We also detected increase in goblet cell number in all infected animal groups compared to uninfected animals (*P* = 0.003). To further valid our observation, we used periodic acid Schiff staining for the visualization of goblet cells. In uninfected mice, goblet cells were distributed among the epithelial cells covering the villi. A significantly higher number of goblet cells were observed in intestine sections from *T. spiralis*-infected animals compared to uninfected animals (*P* < 0.003). There was no significant difference in the number of goblet cells between *A. annua* or albendazole-treated mice and infected untreated mice (Figures 4, 5).

### *A. annua* treatment reduced TGF-β expression in intestinal tissues of *T. spiralis-*infected mice

**Figure 6:**
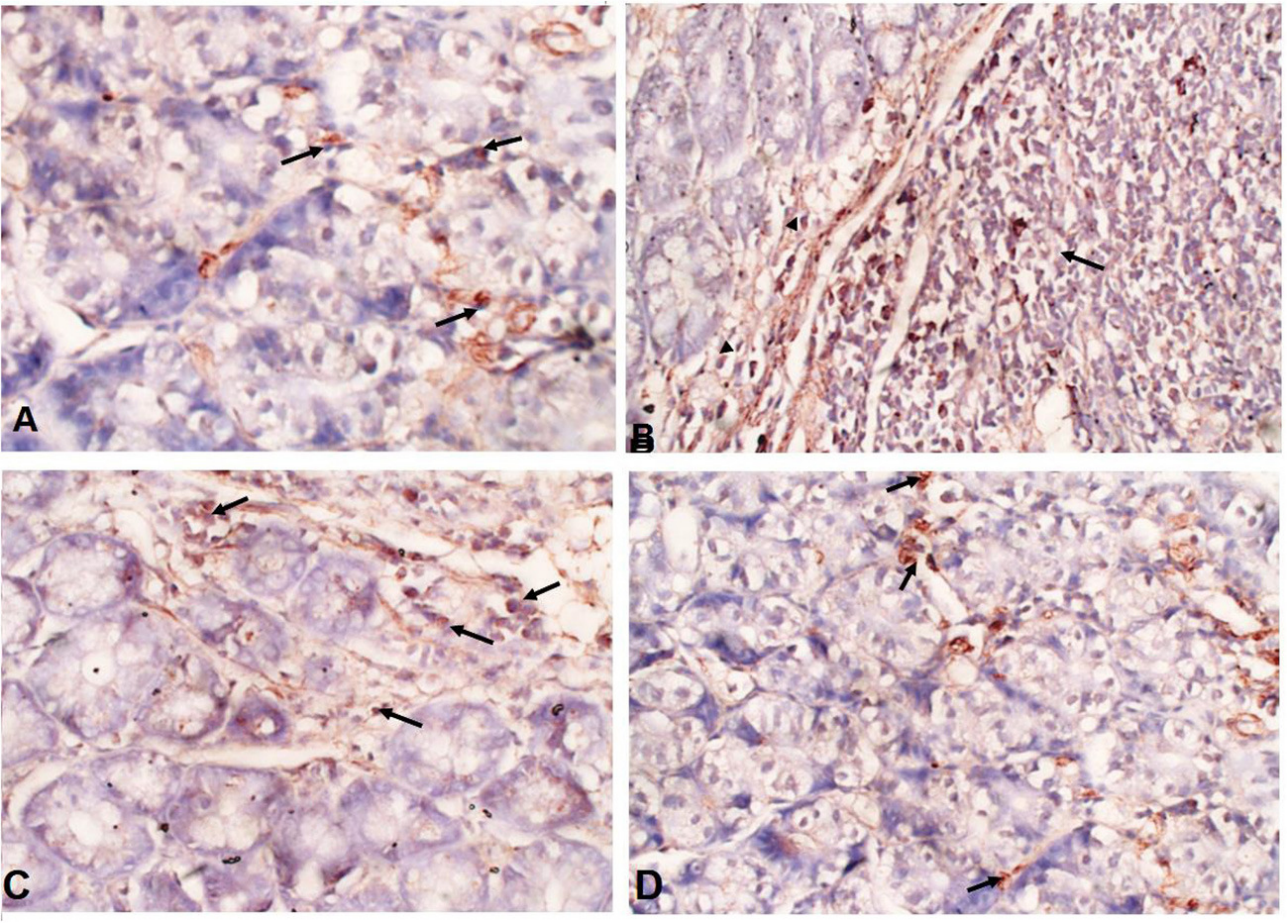
***A***. *annua* treatment markedly reduced TGF-β expression in intestinal tissues of *T. spiralis*-infected mice. (**A**–**D)** Immunohistochemical staining of TGF-β in intestinal tissues of different mice groups **A**. Representative image of intestinal tissue of uninfected mice showing weak expression of TGF-β (black arrows). **(B)** Representative image of intestinal tissue of infected untreated animals showing high expression of TGF-β in submucosal inflammatory cells (black arrows) and mucosal stromal cells (arrow heads). **(C)** Representative image of intestinal tissue of albendazole-treated animals showing reduction of TGF-β-positive mucosal stromal cells (black arrows). **(D)**Representative image of intestinal tissue of *A. annua*-treated mice showing marked reduction in TGF-β -positive mucosal stromal cells (black arrows). Magnification is 200x.

Immunohistochemical staining of the intestinal tissues showed weak expression of *TGF-*β in the intestinal tissues of uninfected mice (black arrows) (Figure 6A). In contrast, elevated expression of TGF-β was detected in mucosal stromal cells (arrow heads) extending to the submucosal inflammatory cells (black arrows) in infected untreated animals (Figure 6B). Interestingly, albendazole and *A. annua*-treated mice showed a significantly lower number of positive cells (black arrows) (35.70 and 20.75%, respectively) (Table 1; Figures 6C,D).

**Table 1:** Percentage (%) of TGF-β positive cells in the intestinal tissue of different groups of mice.

**Groups**	**TGF-β positive cells (%)**
*T. spiralis* uninfected mice	10%
*T. spiralis*-infected untreated mice	80.20%
Albendazole-treated mice	35.70%
*A. annua-*treated mice	20.75%

## Discussion

Trichinellosis is a worldwide parasitic zoonotic disease that is commonly treated with benzimidazole derivatives, including albendazole. Toxicity, adverse effects, low bioavailability, and drug resistance limit the therapeutic utility of those anti-trichinella drugs. Therefore, the development of new and safe alternatives for treatment of trichinellosis is a necessity. Several recent studies have shown that medicinal plants may represent safe and effective alternative therapeutics for treatment of trichinellosis (23, 58–60). In our present study, we aimed to evaluate the efficacy of ethanolic extract of *A. annua* in treating intestinal trichinellosis in experimentally infected mice.

Our results demonstrated that treatment with *A. annua* extract significantly reduced the number of adult worms in the small intestine compared to infected untreated animals. Similarly, Abo Rayia et al. (61) reported 75% reduction in *T. spiralis* adult worm count after treatment of infected mice with artemisinin; the main active constituent of *A. annua*. Furthermore, Caner et al. (18) reported a good therapeutic effect of *A. vulgaris* and *A. absinthium* extracts against the *T. spiralis* larval stages in muscles.

Of note, several studies have shown that *A. annua* is also effective against many parasitic diseases such as trypanosomiasis (62), schistosomiasis (62), toxoplasmosis (34), leishmaniasis (34), giardiasis (33), and coccidiosis (63).

Our data also showed that treatment with *A. annua* ameliorated the *T. spiralis* induced histopathological changes in infected mice. In infected untreated animals, we observed prominent villous atrophy with marked chronic inflammatory cell infiltrates composed of neutrophils, lymphocytes, and macrophages. The administration of *A. annua* and albendazole to *T. spiralis*-infected mice restored the normal villi structure and reduced inflammatory cellular infiltration.

Although our treatments reduced inflammatory cellular infiltration, high eosinophilic infiltration was still recognized in all infected animal groups, including treated animals. We believe that high number of eosinophils in *A. annua*-treated mice was not caused by the treatment since high eosinophils count was noticed in all infected animals including the infected untreated group. Our speculation is supported by previous studies which showed that *A. annua* caused a decline in eosinophils count by 27% when used for treatment of schistosomiasis (64), and suppressed infiltration of mast cells and eosinophils into the epidermis (65), when used in treatment of atopic dermatitis. Moreover, infection with *T. spiralis* has been shown to trigger an increase in eosinophil counts (66). Eosinophils have been reported to play a major role in resistance to parasitic infections, including that of *T. spiralis*, and to kill newborn larvae (NBL) of this parasite (13). Additionally, IL5-dependent eosinophils promote worm expulsion during *T. spiralis* infection (67). The role of eosinophils was further confirmed by a study which detected a rich eosinophil infiltrate in the myocardial tissue of one patient who died of severe trichinellosis (68). In contrast, other studies suggested that eosinophils are essential for protection of NBL. Huang et al. suggested that recruitment of eosinophils to the site of infection may protect NBL by stimulating the production of IL-10 from DCs and CD4+ T cells which inhibit the activation of macrophages and neutrophils (the source of NO) and subsequently prevent NO production, thus promoting *T. spiralis* larval survival (69). This is further supported by the observation that eosinophil-ablated mice cleared *T. spiralis* adult worms normally (70, 71). Despite all of the above findings, the role of eosinophils in host defense against *T. spiralis* or promotion of parasite growth and survival remains to be determined.

Our histological data also demonstrated increased goblet cell counts in the intestinal sections of all infected mice groups compared to uninfected mice. It is well documented that goblet cells have a significant role in protection of the intestinal epithelium from intestinal pathogens by secretion of mucins which form a protective viscous barrier against invading organisms including intestinal nematodes (72, 73). Using PAS, we noticed that the infected untreated and treated animal groups have higher numbers of goblet cells, a phenomenon that usually accompanies the invasion of *T. spiralis* (8, 12, 14, 74, 75). Goblet cells secrete various types of active molecules, including mucin glycoproteins, which are essential for the formation of the mucus overlaying the surface of epithelial cells causing the trapping of the worms and their expulsion from the host (76–78). This has been confirmed by the observation of delayed worm expulsion in Stat6- or IL-4Ra-deficient mice infected with *T. spiralis*, as those mice were not able to develop goblet cell hyperplasia (12, 79). In addition to mucus, TFF3 (trefoil factor) is produced by goblet cells which helps prevent disturbances in the mucosa and improves regeneration (80).

Several reports have discussed the important role of TGF-β in adult-worm expulsion during intestinal trichinellosis (81–83). Therefore, in the present study, we used immunohistochemical staining to quantify the expression of TGF-β in intestinal tissues of *T. spiralis*-infected mice. According to our results, the production of TGF-β was increased in infected untreated mice, whereas lower numbers of TGF-β-positive cells were observed in treated animals. Similar findings were reported by previous studies which support the role of TGF-β in worm expulsion (81–84). Akiho et al. reported that gut contraction during *T. spiralis* infection is driven by Th2 cytokines and TGF-β via STAT6 and COX-2, respectively (85, 86). Moreover, Steel et al. stated that TGF-β is the main player in intestinal contraction and worm expulsion (87). Using mice which lacked the ability to produce TGF-β in experimental intestinal trichinellosis model, they observed that the mice lacked the ability to contract intestinal muscles and flush out the parasite (16, 87).

## Conclusions

We showed that *A. annua* extract eliminated the adult worms from the intestine of *T. spiralis*-infected mice. Moreover, our extract treatment restored the normal intestinal architecture, villi length, and reduced inflammatory cellular infiltration. Moreover, treated mice showed modest expression of TGF-β, which has been proven to play a major role in worm expulsion. The therapeutic effect of *A. annua* extract was comparable to albendazole; however, the side effects and the reported resistance to benzimidazole derivatives, including albendazole support further testing of *A. annua* extract as a promising complementary herbal product for treatment of trichinellosis. More studies are required to investigate the anti-trichinella active components of *A. annua* and to develop more effective and safer derivatives that can be used in combination to limit the development of resistance.

## Data availability statement

The original contributions presented in the study are included in the article/supplementary material, further inquiries can be directed to the corresponding author/s.

## Ethics statement

The animal study was reviewed and approved by Research Ethics Committee of the Faculty of Veterinary Medicine, South Valley University, Egypt (Protocol code 20-4-9-2021).

## Author contributions

All authors contributed to the conceptualization, experimental design, data analysis, writing, review, and editing of the manuscript. Additionally, HE and AE-k led the research team, supervised the study, critically analyzed the data and revised the manuscript. All authors contributed to the article and approved the submitted version.

## Conflict of interest

The authors declare that the research was conducted in the absence of any commercial or financial relationships that could be construed as a potential conflict of interest.

## Publisher's note

All claims expressed in this article are solely those of the authors and do not necessarily represent those of their affiliated organizations, or those of the publisher, the editors and the reviewers. Any product that may be evaluated in this article, or claim that may be made by its manufacturer, is not guaranteed or endorsed by the publisher.
